# Placental volume

**DOI:** 10.1186/1471-2393-12-S1-A4

**Published:** 2012-08-28

**Authors:** Harvey J Kliman

**Affiliations:** 1 Yale University School of Medicine, New Haven, CT, USA

## 

The relationship between placental size and pathology and risk of stillbirth is well recognised. Placental volume may predict birthweight and by association, outcome of pregnancy. The size of the placenta can be determined by two-dimensional sonography and volumetric mathematic modelling [1] and placental volume could be calculated at each ultrasound scan. We have developed a mathematical solution to accurately estimate intrauterine placental volume. Caregivers of pregnant women currently only track the growth of the fetus without any insight into the growth of the placenta, despite its importance in prenatal development. In situations where the placenta is significantly small or large for gestational age, a caregiver may not have any warning that the fetus is compromised or near death until it is too late. Fetal complications due to placental abnormalities occur in as many as 20% of pregnancies. Clinical intervention is possible with early detection. This invention allows for assessment of *in utero* placental volume using three basic measurements: width, height and thickness of the placenta. There are no alternative simple, reliable or convenient methods to determine the volume and/or weight of a placenta prior to delivery available today. We propose to use this method to generate normative data on a large population of pregnant women which can be used to automatically flag abnormal placental size. Such normative data will form the basis for the generation of tables which can be incorporated into future ultrasound devices. This will empower future caregivers to identify and intervene in cases where an intrauterine fetal death would have been the first indication of any problems.
